# Cost-Effectiveness Analysis of a Three-Drug Regimen Containing Bevacizumab for the Treatment of Recurrent Pediatric *Medulloblastoma* in China: Based on a COG Randomized Phase II Screening Trial

**DOI:** 10.3389/fpubh.2022.914536

**Published:** 2022-06-02

**Authors:** Zhaoyan Chen, Fangyuan Tian, Xi Chen

**Affiliations:** ^1^Department of Pharmacy, West China Hospital, Sichuan University, Chengdu, China; ^2^Department of Integrated Care Management Center, West China Hospital, Sichuan University, Chengdu, China

**Keywords:** recurrent pediatric *medulloblastoma*, bevacizumab, *temozolomide*, irinotecan, cost-effectiveness

## Abstract

**Background:**

*Medulloblastoma* is the most common malignant brain tumor of childhood, accounting for 6 to 7 percent of all childhood CNS tumors. The purpose of this study was to evaluate the economic efficacy of a bevacizumab combined with *temozolomide* + irinotecan regimen for the treatment of recurrent pediatric *medulloblastoma* in China.

**Methods:**

The data analyzed were from a randomized phase II screening trial that showed an improved survival benefit in child patients with recurrent *medulloblastoma* treated with a T+I+B combination regimen. A Markov model is constructed to estimate the incremental cost–effectiveness ratio (ICER) from the perspective of Chinese society. The uncertainty in the model is solved by one-way certainty and probabilistic sensitivity analysis.

**Results:**

Our base case analysis showed that the total costs of treatment increased from $8,786.403 to $27,603.420 with the combination bevacizumab vs. the two-agent chemotherapy regimen. Treatment with T+I+B combination therapy was associated with an increase in effectiveness of 0.280 QALYs from 0.867 to 1.147 QALYs T+I regimen. The incremental cost-effectiveness ratio was $67,203.632/QALY, which exceeded our pre-specified willingness-to-pay threshold ($38,136.26/QALY). Cost changes associated with grade 3–4 AE management, tests used, or hospitalization costs had little effect on the ICER values predicted by sensitivity analysis.

**Conclusions:**

Taken together, the results of this study suggest that the combination of bevacizumab with temozolomide and irinotecan is not a cost-effective option from the perspective of Chinese payers as a first-line treatment option for children with recurrent *medulloblastoma* in China.

## Introduction

Medulloblastoma is the most common malignant brain tumor of childhood, accounting for 6 to 7 percent of all childhood CNS tumors (malignant and non-malignant) (1), and occurs in the posterior fossa, mainly in the cerebellum. Most patients are treated with a combination of surgery, radiation therapy (RT), and chemotherapy. Currently, approximately three-quarters of patients survive for a long time, but each treatment modality leads to late complications, which have a great impact on the quality of life of patients. In a retrospective study of 1,485 children with primary CNS tumors who attended a neurosurgery center in China between 2001 and 2005, *medulloblastoma* was ranked third among the top five most common brain tumors (2).

Approximately 30% of children with MB will relapse after aggressive treatment, including surgery and chemotherapy, with or without radiation. Treatment options for recurrent *medulloblastoma* are still controversial and lack standards (3, 4). Tumor-targeted therapy at relapse appears to improve overall survival (OS) compared with palliative care alone, but long-term survival remains below 10% in most studies. Therefore, the results of this largest cohort study to date shed new light on regimen options for children with recurrent *medulloblastoma* (5). The study showed that bevacizumab combined with *temozolomide* + irinotecan regimen can significantly prolong event-free survival and overall survival in children and improve prognosis.

Although bevacizumab combination therapy is effective and well tolerated in children with recurrent *medulloblastoma*, the high cost of these drugs must be considered. These high costs can have profound effects on patients in the form of financial toxicity, causing patients to abandon or delay care, reduce quality of life, and put patients at risk of bankruptcy. From a social point of view, as a country with a large population, China has relatively underdeveloped medical resources, unbalanced regional economic development, and large differences in local medical insurance policies. In recent years, the national oncology drug negotiation agenda has been increasingly advanced, and the pricing of many drugs has undergone great changes. Few studies have examined the economics of bevacizumab in children with recurrent *medulloblastoma*. Therefore, in this context, our study uses the Markov model to evaluate the economics of the T+I+B scheme in China, aiming to provide necessary reference and data support for doctors, patients and policy makers.

## Materials and Methods

### Target Population

Inclusion criteria included patients under the age of 21 who relapsed or were refractory to standard chemotherapy, and the number of relapses was fixed at 1–2. All had a histological diagnosis prior to enrollment, and residual disease was defined as tumor measurable on MRI in two perpendicular diameters. Organ function was also assessed. Enrolled patients were randomized to receive either a two-drug regimen including temozolomide (TMZ, 50 mg/m^2^ PO for 5 days) plus irinotecan (IRT, 50 mg/m^2^ IV for 5 days) or TMZ, IRT plus bevacizumab (BEV, 10 mg/kg IV on days 1 and 15). The regimen was repeated every 28 days for a maximum of 12 courses until intolerable toxicity or disease progression.

### Model Structure

**Figure 1:**
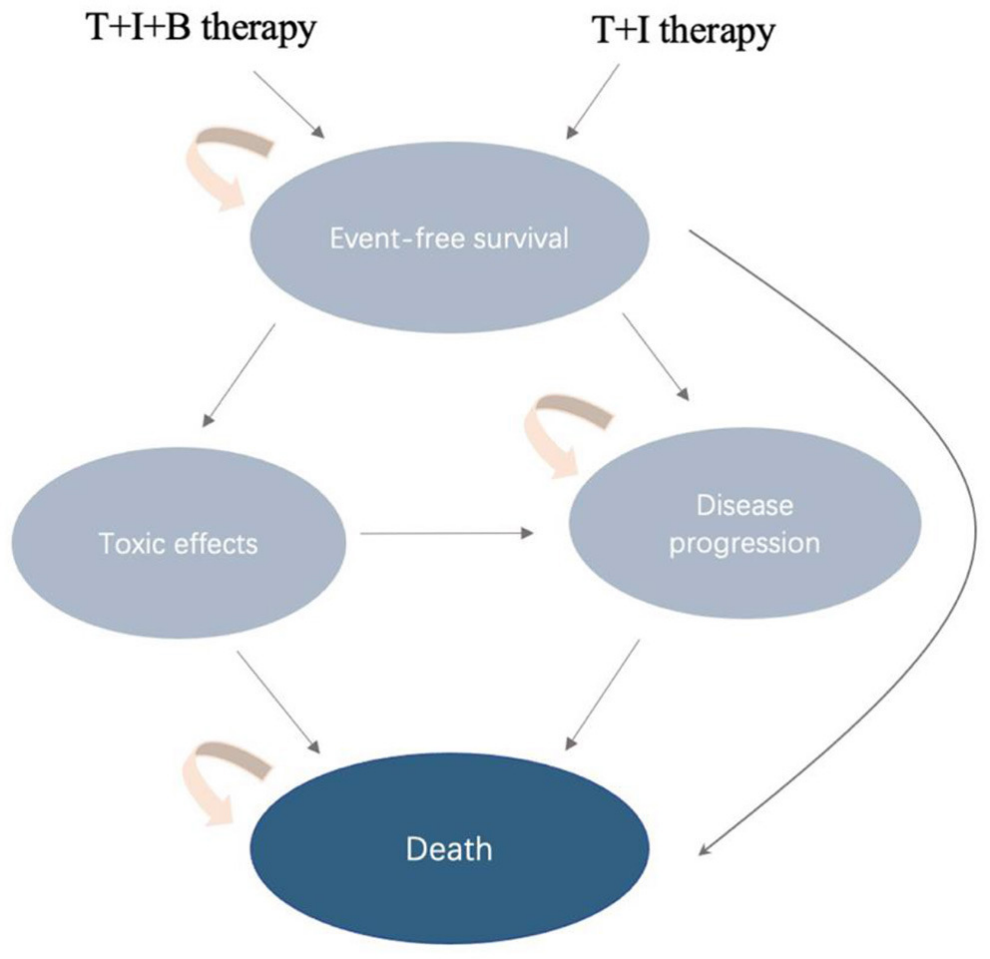
Diagram of transitions between health states.

Patients enter the Markov model in a stable disease state, and then they may remain in a stable disease state (event-free survival, EFS) or experience toxic effects, disease progression (PD), or death (Figure 1). The transition probabilities for these events were derived from COG data. We extracted progression and survival data from reported Kaplan–Meier curves. Similar to previous cost-effectiveness studies, we only included and assessed grade 3 to 4 treatment-related adverse events. Toxicity was defined using the National Cancer Institute Common Toxicity Criteria (version 3.0). According to the survival and follow-up time, we set the model period to be 1 month. We reconstructed individual patient data through R software, and the transition probability was estimated through the reported survival curve. The standard for setting the running time of the model is that 99% of patients enter the termination state. The time horizon chosen for this model is 10 years.

### Model Parameters

Costs are estimated from the perspective of Chinese society (Table 1). The following costs were considered during the analysis: all medications, tests (MRI, biochemistry, etc.), management of grade 3–4 adverse events (AEs), and hospitalization. In view of the fact that hidden costs are often difficult to be accurately counted in real life, and there are large individual differences, the hidden costs of this study were not included in the calculation. For the drug dose parameters, we used the weighted average method to estimate height and weight with reference to the latest survey results of Chinese children (9). The formula for calculating the body surface area (BSA) of children is BSA(m^2^) = 1.05+ [body weight (kg) – 30(kg)] 0.02(m^2^). The unit price of each drug and examination is based on the 2022 charging standard of West China Hospital of Sichuan University and the winning bid price in the market. We estimated the cost of second-line treatment for both groups of patients based on survival data reported in the trial by Leary et al. (6). All fees are converted at RMB 6.437 per USD (March 2022). Health outcome data in this model were obtained from a randomized, controlled study. Survival time was expressed in quality-adjusted life years (QALY). Since basic information on utility value was not mentioned in the original literature, health utility value was referred to in published studies (7). Utility values for event-free survival, disease progression, and death status were 0.89, 0.73, and 0.00, respectively. The model parameters related to cost and benefit are shown in Table 1. According to the recommendations of the 2020 China Pharmacoeconomic Evaluation Guidelines and the Handbook (8), the cost and utility values were discounted at an annual discount rate of 3%, and a sensitivity analysis was performed.

**Table 1:** Parameters for the base case cost-effectiveness model.

**Parameters**	**Value**	**Distribution**	**Source**
**Clinical efficacy, months**			
Median EFS			
T+I+B	9	Weibull	(5)
T+I	6	Weibull	(5)
Median OS			
T+I+B	19	Weibull	(5)
T+I	13	Weibull	(5)
**Drug costs per cycle, $**			
Conbination T+I+B	2803.73	Gamma	Listed price
Conbination T+I	919.90	Gamma	Listed price
Temozolomide in T+I+B regimen	343.75	Gamma	Listed price
Irinotecan in T+I+B regimen	576.15	Gamma	Listed price
Bevacizumab	1,883.83	Gamma	Listed price
Temozolomide in T+I regimen	343.75	Gamma	Listed price
Irinotecan in T+I regimen	576.15	Gamma	Listed price
Second-line treatment in T+I+B regimen	233.11	Gamma	(6)
Second-line treatment in T+I regimen	305.67	Gamma	(6)
Hospitalization costs in T+I+B regimen	43.96	Gamma	HIS
Hospitalization costs in T+I regimen	43.96	Gamma	HIS
**Drug toxic effects costs, $**			
T+I+B	10.09	Gamma	(5), Listed price
T+I	13.87	Gamma	(5), Listed price
**Tests costs per cycle, $**			
T+I+B	185.63	Gamma	(5), HIS
T+I	176.56	Gamma	(5), HIS
**Disease costs per cycle, $**			
Event-free survival in T+I+B	3,043.40	Gamma	Listed price, HIS
Event-free survival in T+I	1,154.28	Gamma	Listed price, HIS
**Disease status utility per year, QALY**			
Event-free survival	0.89	Beta	(7)
Progressed disease	0.73	Beta	(7)
Death	0.00	Beta	(7)
**Discount rate, %**	3.00	Beta	(8)

*HIS, Hospital Information System; EFS, Event-free Survival; OS, Overall Survival; T, Temozolomide; I, Irinotecan; B, Bevacizumab; QALY, Quality-Adjusted Life Year*.

### Statistical Analysis

Cost-effectiveness was measured using an incremental cost-effectiveness ratio (ICER), which is the ratio of the differences in cost (measured in US dollars) and effectiveness (measured in QALYs) between the 2 treatments. We adopted a willingness-to-pay threshold of 3 times China's GDP per capita ($38,136.26 per QALY), which is considered cost-effective if ICERs are below $38,136.26 per QALY. We performed 1-way deterministic sensitivity analyses of each variable in the model to evaluate which variables had the greatest consequences for cost-effectiveness. The variation range of the unit price of the drug refers to the winning price of the drug announced on the official websites of different provinces and cities. To further assess model uncertainty, we performed a probabilistic sensitivity analysis using a Monte Carlo simulation with 1,000 repetitions, allowing us to simultaneously vary uncertainty in cost, health utilities, and transition probabilities.

## Results

### Base-Case Analysis

**Figure 2:**
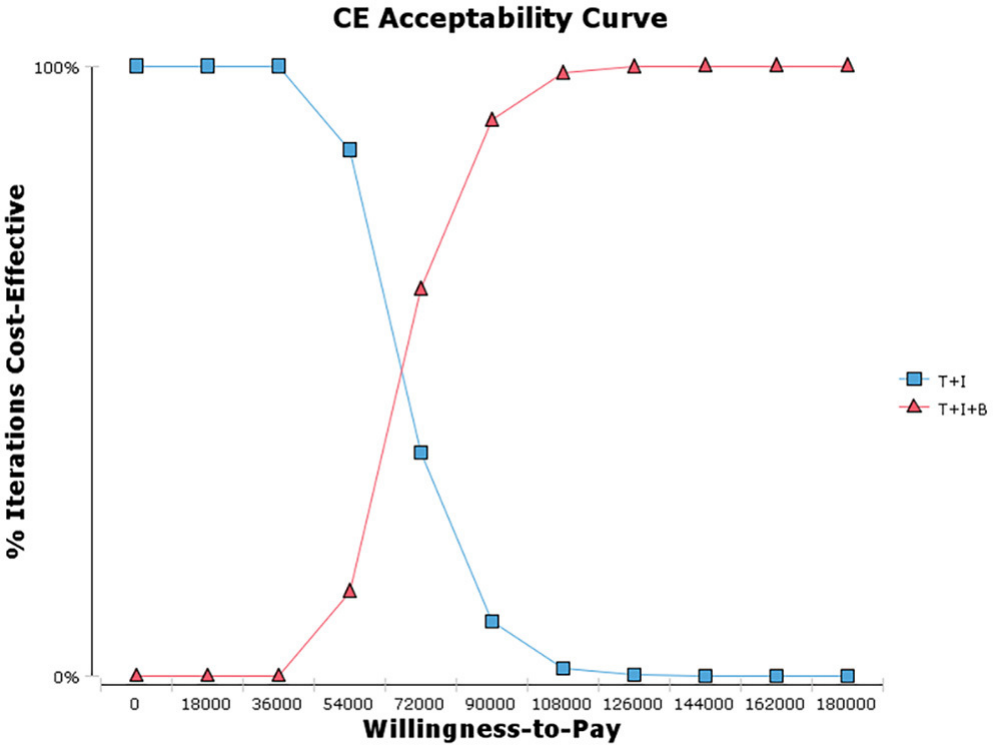
Cost-effectiveness acceptability curves.

Our base case analysis showed that the total costs of treatment increased from $8,786.403 to $27,603.420 with the combination bevacizumab vs. the two-agent chemotherapy regimen. Treatment with T+I+B combination therapy was associated with an increase in effectiveness of 0.280 QALYs from 0.867 to 1.147 QALYs T+I regimen. The incremental cost-effectiveness ratio was $67,203.632/QALY, which exceeded our pre-specified willingness-to-pay threshold ($38,136.26/QALY) (Figure 2, Table 2). Considering the increased total cost, combination therapy with *temozolomide*, irinotecan, and bevacizumab is not an economical treatment option for children with recurrent *medulloblastoma* unless there is an appropriate grant program and health insurance policy support.

**Table 2:** The results of the cost-effectiveness analysis.

**Parameters**	**T+I+B**	**T+I**
cEFS	25,598.444	6,543.936
cPD	2,004.976	2,242.467
uEFS	0.624	0.421
uPD	0.523	0.446
Total costs	27,603.420	8,786.403
Total effectiveness	1.147	0.867
Incremental costs	18,817.017	**/**
Incremental effectiveness	0.280	**/**
Total C/E	24,065.754	10,134.260
ICER $/QALY	67,203.632	**/**

### Sensitivity Analysis

**Figure 3:**
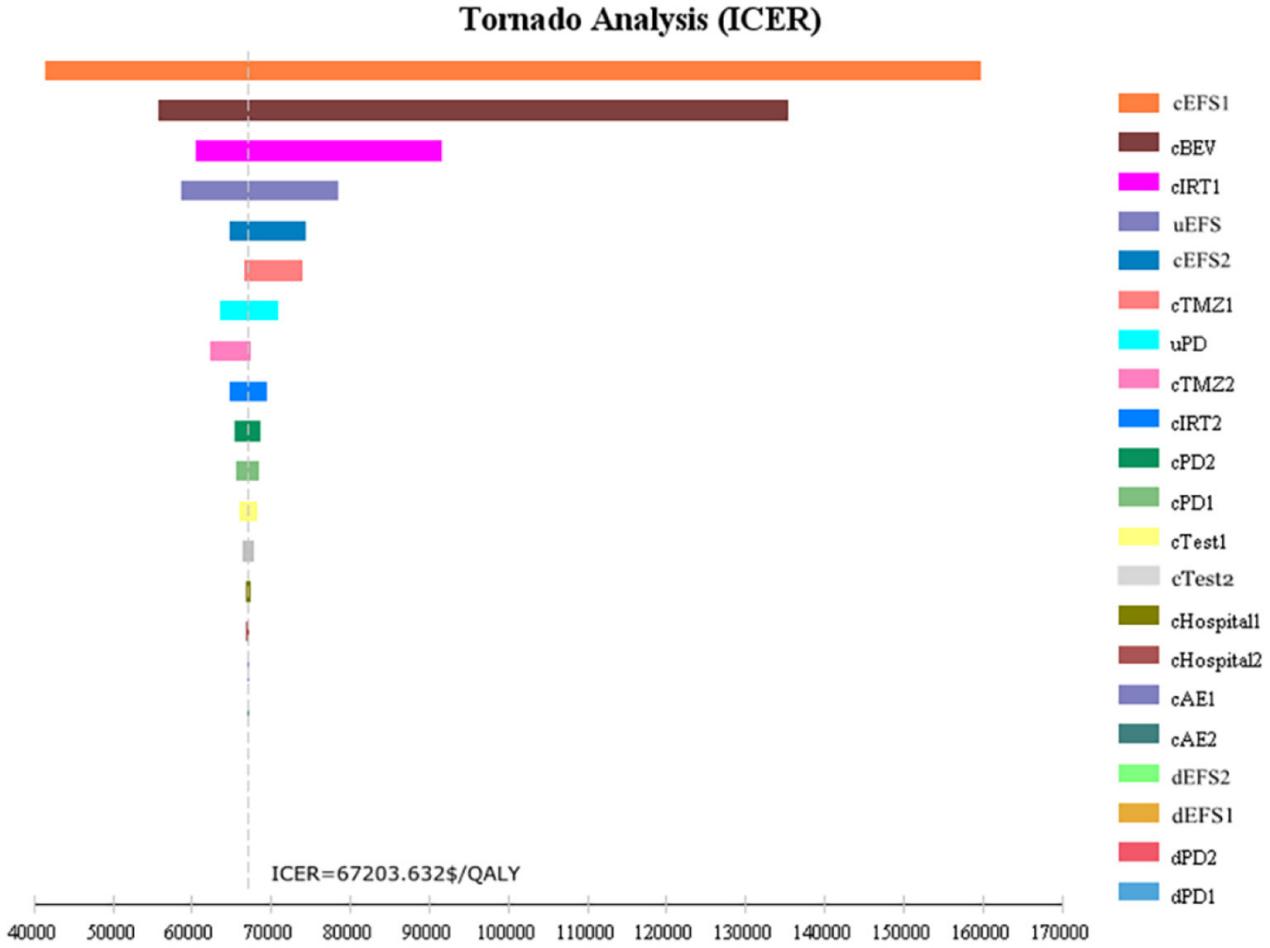
Tornado diagram of one-way sensitivity analysis.

**Figure 4:**
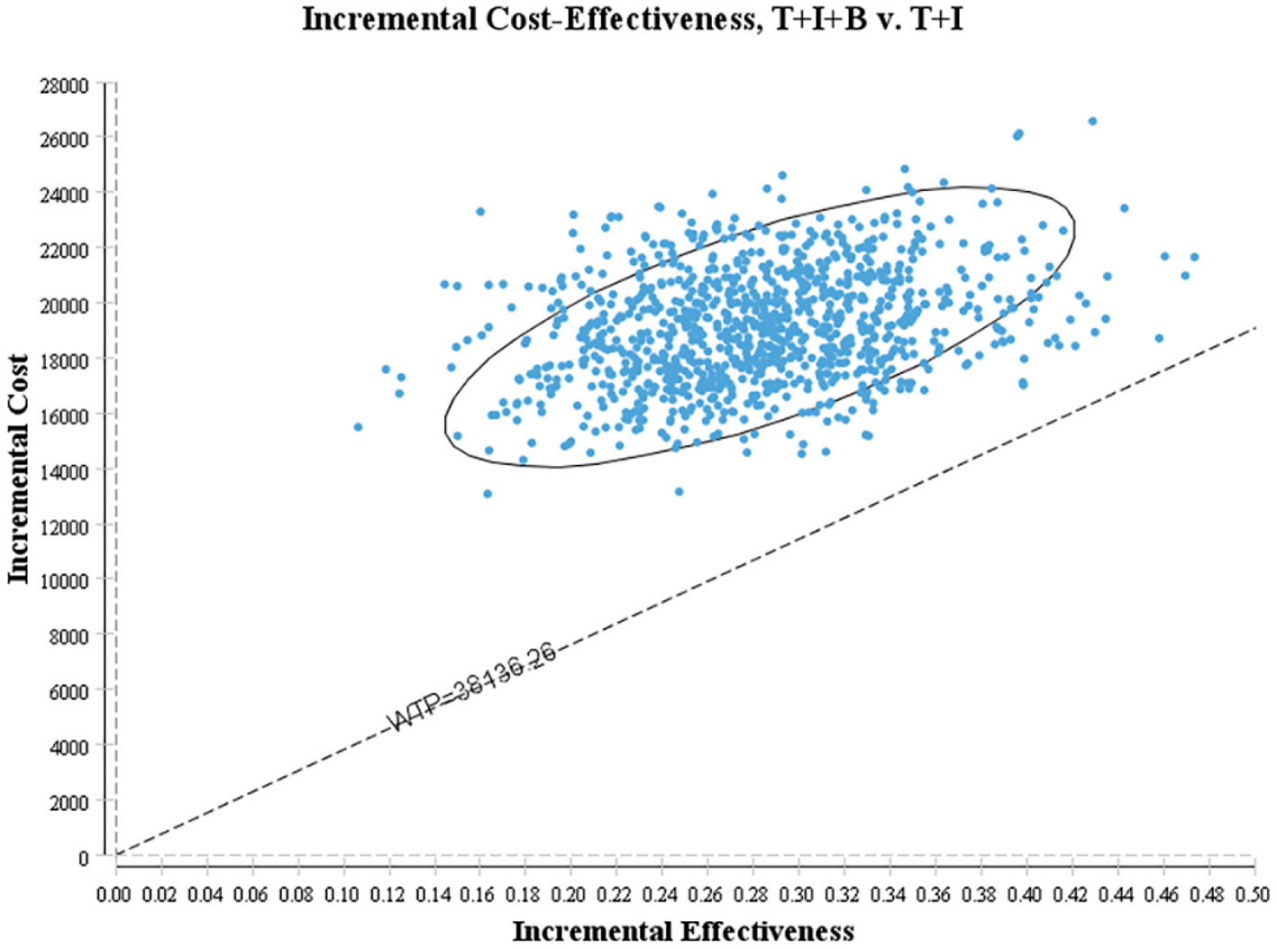
Scatter plot of probabilistic sensitivity analysis.

One-way sensitivity analysis was conducted to assess the impact of individual parameters in the Markov model. The results are illustrated using a tornado diagram (Figure 3). The costs of EFS state for the T+I+B group, costs of bevacizumab, and costs of irinotecan in the T+I+B group were the most influential parameters of the Markov model. In a univariate sensitivity analysis, the three-drug combination only decreased when the monthly drug cost of BEV decreased from $1,883.83 to $916.19 (a 51.4% reduction) or when the monthly combined cost of EFS status decreased from $3,042.40 to $2,075.60 (a 31.7% reduction). The T+I+B treatment regimen became economical at a willingness-to-pay threshold of $38,136.26/QALY. However, variations in the costs related to the management of grade 3–4 AEs, tests used or hospital fees incurred had a smaller impact on the ICER values predicted by sensitivity analysis. Additionally, probabilistic sensitivity analysis (1,000 iterations) demonstrated that the ICER was consistently greater than $38,136.26/QALY (Figure 4).

## Discussion

In this cost-effectiveness study, we found that bevacizumab combination therapy cannot be considered a cost-effective first-line regimen for children with recurrent *medulloblastoma* compared with dual-agent chemotherapy. Our model is not particularly sensitive to hospitalization costs or treatment costs for toxic effects. Notably, our model found that only when the monthly drug cost of BEV decreased from $1,883.83 to $916.19 (a 51.4% reduction) or when the monthly combined cost of EFS status decreased from $3,042.40 to $2,075.60 (a 31.7% reduction). The T+I+B treatment regimen became economical at a willingness-to-pay threshold of $38,136.26/QALY.

Cost-effectiveness analysis of bevacizumab for various types of brain tumors has been reported. In an economic review of bevacizumab for first-line treatment of newly diagnosed glioblastoma multiforme, the addition of bevacizumab to radiation therapy and temozolomide resulted in 0.13 quality-adjusted life years (QALY), and patients with an $80,000 increase in treatment cost over a 2-years time frame had a 0% probability of being cost-effective at a willingness-to-pay threshold of $100,000/QALY (10). In addition, bevacizumab has also shown some efficacy in metastatic solid tumors. A study of metastatic colorectal cancer in the United States showed that the total cost of capecitabine and bevacizumab needs to be reduced from $6,173 to $452, and it would be cost-effective at the willingness-to-pay threshold at the median US household income ($59,039/QALY) (11). In 2021, bevacizumab and atezolizumab significantly improved progression-free survival (PFS) and overall survival in patients with liver cancer in the IMbrave 150 trial compared with sorafenib alone. Total utility has increased by 0.53QALY, but its economics have not been shown in either China (WTP = $28,527.00/QALY) or the US (WTP = $150,000.00/QALY) market environments.

Bevacizumab is an anti-vascular endothelial growth factor humanized recombinant monoclonal IgG1 antibody that was approved by the US FDA in 2007. Its mechanism is to block the binding of vascular endothelial growth factor to its receptors and inhibit the promotion of vascular endothelial growth factor. Generate activity, thereby exerting an antitumor effect (12). In recent years, it has been found that bevacizumab may weaken the resistance of tumors to traditional chemotherapeutic drugs. The main reasons include increasing the blood concentration of chemotherapeutic drugs, prolonging the half-life of chemotherapeutic drugs, reducing the pressure of tumor interstitial fluid, and facilitating chemotherapeutic drugs to reach the tumor site (13). However, there are few large multicenter randomized controlled studies on bevacizumab in children's brain tumors. Adam et al. found that bevacizumab combined with temozolomide and irinotecan can significantly prolong the treatment of children with 8 years of follow-up. The event-free survival time and overall survival time of 10 years through the Markov model showed that the total utility increased by 0.28QALY, but it also brought a total increase of $18,817.017/person in treatment costs.

We considered the dose difference between children of different races in the model design and converted it through the body surface area formula. Toxicity profiles were comparable in both treatment arms in the trial. We still considered the cost of drug toxicity treatment. In view of the unclear social division of labor among children, the cost of lost work is not included in the calculation. It is worth noting that, as a developing country, China has a vast territory, uneven regional development, and a large economic gap between coastal and inland economies. With the advancement of medical and health reform, tumor drugs frequently appear in the national medical insurance negotiation catalog, and drug prices fluctuate greatly. Therefore, we investigated the winning bid prices in representative areas of China, east, west, north and south, and included the median in the sensitivity analysis to evaluate the stability of the model.

Univariate sensitivity analysis found that the cost of EFS status in the T+I+B group and the cost of bevacizumab and irinotecan in the T+I+B group were the most influential parameters of the Markov model. The three-drug combination decreased only when the monthly drug cost for BEV decreased from $1,883.83 to $916.19 (51.4% decrease) or the combined monthly cost for EFS status decreased from $3,042.40 to $2,075.60 (31.7% decrease). The T+I+B regimen became economical at a willingness-to-pay threshold of $38,136.26/QALY.

Our study developed a Markov decision tree model to simulate the process of disease. However, the following limitations still exist: the cost–benefit analysis model is based on phase II clinical trials rather than real-world studies, and the extrapolation of the data has certain limitations; given the lack of reporting of the original study data, the transition probability was estimated, although it has been carried out. We have conducted a single factor sensitivity analysis on the model parameters, but we do not rule out other factors that affect the model. Since the original study did not report the health utility value of children in different disease states, we refer to the published literature related to brain tumors.

Taken together, the results of this study suggest that the combination of bevacizumab with *temozolomide* and irinotecan is not a cost-effective option from the perspective of Chinese payers as a first-line treatment option for children with recurrent *medulloblastoma* in China. However, appropriate drug donation programs and social assistance should be encouraged to make this rare patient population more affordable and improve quality of life.

## Data Availability Statement

The original contributions presented in the study are included in the article/supplementary material, further inquiries can be directed to the corresponding author/s.

## Author Contributions

ZC conceived and designed the experiments, wrote the manuscript, and revised the work critically for important intellectual content. ZC and FT performed the experiments and data analysis. XC and FT provided the reagents, materials and analysis tools. All authors have read and approved the final version of the manuscript.

## Conflict of Interest

The authors declare that the research was conducted in the absence of any commercial or financial relationships that could be construed as a potential conflict of interest.

## Publisher's Note

All claims expressed in this article are solely those of the authors and do not necessarily represent those of their affiliated organizations, or those of the publisher, the editors and the reviewers. Any product that may be evaluated in this article, or claim that may be made by its manufacturer, is not guaranteed or endorsed by the publisher.
